# Learning Analytics Applied to Clinical Diagnostic Reasoning Using a Natural Language Processing–Based Virtual Patient Simulator: Case Study

**DOI:** 10.2196/24372

**Published:** 2022-03-03

**Authors:** Raffaello Furlan, Mauro Gatti, Roberto Mene, Dana Shiffer, Chiara Marchiori, Alessandro Giaj Levra, Vincenzo Saturnino, Enrico Brunetta, Franca Dipaola

**Affiliations:** 1 Department of Biomedical Sciences Humanitas University Milan Italy; 2 IRCCS Humanitas Research Hospital Rozzano, Milan Italy; 3 IBM Active Intelligence Center Bologna Italy; 4 Department of Medicine and Surgery University of Milano-Bicocca Milan Italy; 5 IBM Research Zurich Switzerland

**Keywords:** clinical diagnostic reasoning, learning analytics, natural language processing, virtual patient simulator, medical education, medical knowledge

## Abstract

**Background:**

Virtual patient simulators (VPSs) log all users’ actions, thereby enabling the creation of a multidimensional representation of students’ medical knowledge. This representation can be used to create metrics providing teachers with valuable learning information.

**Objective:**

The aim of this study is to describe the metrics we developed to analyze the clinical diagnostic reasoning of medical students, provide examples of their application, and preliminarily validate these metrics on a class of undergraduate medical students. The metrics are computed from the data obtained through a novel VPS embedding natural language processing techniques.

**Methods:**

A total of 2 clinical case simulations (tests) were created to test our metrics. During each simulation, the students’ step-by-step actions were logged into the program database for offline analysis. The students’ performance was divided into seven dimensions: the identification of relevant information in the given clinical scenario, history taking, physical examination, medical test ordering, diagnostic hypothesis setting, binary analysis fulfillment, and final diagnosis setting. Sensitivity (percentage of relevant information found) and precision (percentage of correct actions performed) metrics were computed for each issue and combined into a harmonic mean (F_1_), thereby obtaining a single score evaluating the students’ performance. The 7 metrics were further grouped to reflect the students’ capability *to collect* and *to analyze* information to obtain an overall performance score. A methodological score was computed based on the discordance between the diagnostic pathway followed by students and the reference one previously defined by the teacher. In total, 25 students attending the fifth year of the School of Medicine at Humanitas University underwent test 1, which simulated a patient with dyspnea. Test 2 dealt with abdominal pain and was attended by 36 students on a different day. For validation, we assessed the Spearman rank correlation between the performance on these scores and the score obtained by each student in the hematology curricular examination.

**Results:**

The mean overall scores were consistent between test 1 (mean 0.59, SD 0.05) and test 2 (mean 0.54, SD 0.12). For each student, the overall performance was achieved through a different contribution in collecting and analyzing information. Methodological scores highlighted discordances between the reference diagnostic pattern previously set by the teacher and the one pursued by the student. No significant correlation was found between the VPS scores and hematology examination scores.

**Conclusions:**

Different components of the students’ diagnostic process may be disentangled and quantified by appropriate metrics applied to students’ actions recorded while addressing a virtual case. Such an approach may help teachers provide students with individualized feedback aimed at filling competence drawbacks and methodological inconsistencies. There was no correlation between the hematology curricular examination score and any of the proposed scores as these scores address different aspects of students’ medical knowledge.

## Introduction

### Background

Virtual patient simulators (VPSs) are didactical tools that require students to face a variety of clinical scenarios. Providing students with software-based medical training that may be integrated with in-person clinical internships can help them develop diagnostic skills [1-8]. Furthermore, through adequate metrics obtained from the analyses of the user’s logged actions, VPSs may generate a multidimensional representation of the students’ medical competence, thus providing teachers with potentially valuable didactical information [9-13]. VPSs may include the use of natural language processing (NLP) techniques to better mimic physician–patient interactions and facilitate the use of these techniques by medical school students [13-15].

In many VPSs, metrics are set up to merely assess sectorial aspects of the overall patient’s diagnostic management, such as history taking [14] or clinical examination [13], whereas, in other VPSs, crucial diagnostic activities such as conducting a physical examination and ordering medical tests are not considered [15]. Therefore, many VPSs and their relative metrics aim to address specific didactical items rather than embracing the overall clinical diagnostic approach. The latter is crucial in undergraduate medical training as most diagnostic errors made by junior physicians are caused by flaws in data collection or data integration [15]. There is a need for novel VPSs that target all areas of the diagnostic process while maintaining the user-friendly features provided by NLP techniques.

In addition to VPSs, another technology that may potentially benefit medical education is the intelligent tutoring system (ITS) [9-13] as it provides students with ad hoc feedback on a step-by-step basis and provides proper remediation suggestions [16,17]. For example, the CIRCISM-Tutor [18] was created to teach first-year medical students blood pressure regulation concepts. The COMET algorithm [19] was applied to problem-based learning by incorporating multimodal interfaces with text and images. The StoichTutor [20] helped students learn stoichiometry, although its application was mostly restricted to high school teaching. From a didactical standpoint, these tools proved to be effective in helping students improve their skills by facilitating reasoning and promoting cognitive associations during the learning process [9-13,21,22]. However, in these cases, ITS technology was not applied to the entire clinical case simulation.

We recently developed a VPS, Hepius, which integrates ITS components [23] that address 2 main activities carried out by a physician when managing a patient: data gathering and data analysis. NLP techniques were used to mimic physician–patient interactions. Data gathering comprised four main components: (1) examination of patient information (ie, the input scenario) in a simulated electronic medical record, (2) medical history collection, (3) physical examination, and (4) diagnostic test order. The data analysis model entailed four main components: (1) hypothesis generation, (2) binary analysis, (3) pattern analysis, and (4) final diagnosis. Student data gathering and analysis performance were addressed and quantified by setting appropriate metrics and general learning analytics.

### Objective

In this study, we describe the learning analytics obtained by tracking medical students’ execution of 2 virtual patient simulations using Hepius. In particular, the results obtained from a group of fifth-year students attending the Humanitas University Medical School are presented and discussed in relation to their potential learning implications. Learning analytics obtained from the first simulation test are also preliminarily confronted with the scores obtained by the medical students on their hematology final examination.

## Methods

### Ethics Approval

In keeping with our Internal Review Board policy at Comitato Etico Indipendente IRCCS- Istituto Clinico Humanitas no ethics approval was applied for because this is a pedagogical research study, not a clinical study. Data were properly anonymized and informed consent was obtained from all participants at the time of original data collection. Finally, the study does not involve any potential risk of damage to the participants and is not associated with any side effect. A simple written communication was sent to the Internal Review Board, as requested.

### Diagnostic Process Simulator Components

This section provides a synthetic description of the main features underlying Hepius’s diagnostic model, which is necessary for the full comprehension of the learning analytics. A detailed description of the program is provided elsewhere [23].

#### Input Scenario

The student is provided with a brief text describing the patient’s current complaint. In this phase, the student is expected to identify the relevant diagnostic factors contained in the text. A diagnostic factor is a piece of defined clinical information that may help reach a diagnosis (eg, the patient has a fever or Blumberg sign is positive).

#### Medical History Collection

The student must collect further diagnostic factors by formulating questions as though interviewing a real patient. The Hepius NLP algorithm pipeline examines the input question and searches for matching answers (if any) in the question set prepared by the simulation author (ie, the teacher). If a match is found, the program displays the simulation question along with the corresponding answer. For example, if the student were to type *Do you have shortness of breath?* in the free-text dialog box, the NLP pipeline would look for a matching question in the simulated case database (eg, *Do you have dyspnea?*) and automatically provide the corresponding answer (eg, *Yes, I have*). This advanced NLP algorithm takes advantage of a previous NLP algorithm developed by our group to automatically identify patients with syncope from an administrative database [24].

#### Physical Examination

The student is requested to understand which physical examinations are relevant for that specific clinical case. The student has the possibility to either select from a drop-down menu or type in appropriate physical examinations in a free-text dialog box. The relevant examinations that should be performed have been previously determined by the simulation author. All relevant and irrelevant actions performed by the students can be tracked and measured.

#### Medical Test Request

The student may choose to order a diagnostic test. The task of requesting a test is performed in the same manner as the physical examinations. A test request is considered correct only if deemed relevant by the simulation author. When correct, the results of the test are provided.

#### Diagnostic Hypothesis

On the basis of the information collected during the previous phases, the student is expected to formulate 1 or multiple diagnostic hypotheses. This is done by inserting the hypothesis in natural language into a free-text dialog box. The NLP component of Hepius is responsible for matching the hypothesized diagnosis with the one selected by the simulation author as the most relevant hypothesis. This NLP component matches the student’s description with the standard Systematized Nomenclature of Medicine–Clinical Terms (SNOMED–CT) description [25] that is saved in the simulation database. If the hypothesis formulated by the student exists in the list of reasonable diagnostic hypotheses set by the author, positive feedback is given, and the diagnostic hypothesis appears in the binary analysis.

#### Binary Analysis

The student is required to make correlations between all the identified diagnostic factors and the diagnostic hypotheses to improve the capability to analyze the gathered information and form connections. For each pair of diagnostic factor–diagnostic hypothesis relations, the student must decide whether a single diagnostic factor increases, decreases, or neither increases nor decreases (ie, it is neutral) the probability of that diagnostic hypothesis. The binary analysis is a simplified form of the script concordance test (SCT) with a Likert scale of only 3 values (1,0, and −1) rather than the standard 5 values, called “anchor descriptors” [26]. Indeed, in the binary analysis, *increase*, *decrease*, and *neutral* act as anchor descriptors in a classical SCT [26,27]. For example, the student is expected to set the binary analysis between the diagnostic factor *Body temperature is 38 °C* and the diagnostic hypothesis *Pneumonia* as *I* (ie, increase). Any other input would be considered a mistake.

Importantly, one of the key differences between classical SCT and Hepius’s binary analysis is that diagnostic factors and diagnostic hypotheses are not provided a priori but, instead, must be formulated by the students. This requires an active reflective process by the learner, which has an inherent educational value. A more detailed discussion of the differences between these 2 educational tools can be found in Multimedia Appendix 1 [23,26,28-32].

#### Pattern Analysis

In this section, a graph is automatically created to represent the binary analysis. The graph shows the diagnostic factor and diagnostic hypothesis nodes. An edge is created whenever the diagnostic factor and diagnostic hypothesis are increase-related or decrease-related according to the binary analysis. The graph is automatically converted into a cognitive fuzzy map [28,33] that displays an associated numerical weight for each node and edge. The student can modify the weight of diagnostic factor–diagnostic hypothesis edges according to their estimated importance of a specific diagnostic factor supporting the likelihood of a certain disease. The effect of such an action is visualized as a corresponding increase or decrease in the dimension of the diagnostic hypothesis node (Multimedia Appendix 1). This provides the student with immediate feedback.

#### Final Diagnosis

In this final step, the student must choose the final diagnosis among the list of diagnostic hypotheses; namely, the one characterized by the greatest probability of being correct.

### Learning Analytics With Hepius

Learning analytics are used to improve and gain insights into learning processes by collecting, analyzing, and interpreting student-generated data [34]. Whenever a student performs an action with Hepius, the action is logged in the program database. As the simulation author (ie, the teacher) has explicitly specified what is the correct action, it is possible through analysis of the simulation execution logs to construct a detailed representation of the student’s performance.

From this detailed representation, we computed synthetic metrics that provide *remedial insights* into the students’ current capability to apply their competencies. By *remedial*, we mean that the insights may be used by the student, teacher, or other stakeholders to improve learning and teaching processes.

### Test Descriptions

We conducted 2 clinical case simulations (tests) with Hepius to set our metrics. Test 1 (April 12, 2018) included 25 students participating in the Patient Management course (fifth year of the School of Medicine) at Humanitas University. The students performed a simulation on a virtual patient whose chief complaint was dyspnea, and the correct final diagnosis was pleural effusion secondary to Hodgkin lymphoma. Test 2 (May 21, 2018) included 36 students of the same course who performed a simulation on a patient who presented with abdominal pain, and the final diagnosis was acute cholecystitis.

All participants were familiar with the use of the program, were instructed to work independently, and had no time limit. In both tests, all actions performed were logged and subsequently analyzed.

### Learning Metrics

Overall, the students’ performance was split into seven sections, which included: (1) the identification of relevant information within the given clinical scenario, (2) history taking (ie, anamnesis), (3) performing a physical examination, (4) ordering medical tests, (5) formulating diagnostic hypotheses, (6) completing a binary analysis by matching the clinical data obtained throughout the simulation with the differential diagnosis, and (7) making the final diagnosis. For each section, we computed a sensitivity metric (ie, how much of the relevant information contained in each section the student was able to find) and a precision metric (ie, how many actions performed by the student were considered correct). These 2 components were combined with a harmonic mean (F_1_), yielding a single score between 0 and 1 (1=perfect sensitivity and precision). This score was used as an index of the student’s performance for each section (Table 1).

**Table 1:** Section metric description.

Section	Sensitivity metric	Precision metric	Section metric description
Input scenario	Percentage of DFs^a^ identified out of all the DFs present in the input scenario	Percentage of DFs identified in the text out of all the text selections performed by the student	Performance in identifying DFs present in the input scenario without selecting nonrelevant text
Anamnesis	Percentage of relevant anamnestic questions identified out of all the relevant anamnestic questions present in the simulation	Percentage of relevant anamnestic questions out of all the questions asked by the student	Performance in asking all the relevant questions without asking superfluous questions
Physical examination	Percentage of relevant physical examinations performed out of all the relevant physical examinations present in the simulation	Percentage of relevant physical examinations performed out of all the physical examinations performed by the student	Performance in carrying out all the relevant physical examinations without carrying out superfluous physical examinations
Medical test	Percentage of relevant medical tests requested out of all the relevant medical tests present in the simulation	Percentage of relevant medical tests requested out of all the medical tests requested by the student	Performance in requesting all the relevant medical tests without asking for superfluous medical tests
DH^b^	Percentage of reasonable DHs identified out of all the reasonable DHs present in the simulation	Percentage of reasonable DHs identified out of all the DHs formulated by the student	Performance in identifying all the reasonable DHs without formulating inappropriate DHs
BA^c^	Percentage of BA mappings correctly executed on the first attempt out of the total number of BA mappings present in the simulation	Percentage of BA mappings correctly executed on the first attempt out of the total number of BA mappings executed by the student	Performance in identifying the correct DF–DH relationships (increase, neutral, and decrease) on the first attempt
Final diagnosis	Percentage of correct diagnoses identified by the student out of the total number of correct diagnoses present in the simulation	Percentage of correct diagnoses identified by the student out of the total number of diagnoses (correct and incorrect) formulated by the student	Performance in identifying the correct final diagnoses

^a^DF: diagnostic factor.

^b^DH: diagnostic hypothesis.

^c^BA: binary analysis.

By combining the 7 F_1_ metric scores, we obtained a single number that was used as the student’s overall score and compared it with the average class performance.

In addition, the 7 metrics were divided into two groups: one representing the capability to collect information (items 1, 2, 3, and 4) and the other representing the capability to analyze it (items 5, 6, and 7). The choice of developing an accuracy-based metric to assess performance in clinical data gathering rather than simply increasing a cumulative score whenever new information was obtained stemmed from the vast literature supporting the concept that good diagnosticians perform focused data gathering, primarily according to “illness scripts” [35-39]. In other words, this metric aims to measure quality rather than quantity of the collected clinical data.

In addition, for every simulation, the results were depicted on a radar chart. This provided a synthetic view of single student and mean class performance in each of the exercises. Individual radar charts can be superimposed and therefore compared with those achieved by the class.

In virtual patient simulations such as in real-life clinical cases, the proper sequence of diagnostic actions is often crucial for proper diagnosis [40]. In Hepius, these actions are defined as critical diagnostic acts and, when performed according to the expected execution order, they constitute the *desired execution path*. Thus, it is possible not only to analyze whether all crucial diagnostic acts were performed but also if their order was in keeping with the desired execution path. This is synthesized by an additional metric, the *methodological score*, which evaluates the overall diagnostic process [41-43].

To compute the methodological score, the sequence of crucial diagnostic acts performed by a student is converted into a string where each character represents a specific simulation section. The string is then simplified by removing the repetitions of contiguous identical characters. Hence, if the student first identifies 3 scenario factors, then asks 2 anamnestic questions, and, finally, executes 2 physical examinations, this would be initially converted into the string *sssaapp*. In such a string, *s* stands for scenario, *a* for anamnesis, and *p* for physical examination. This string would be further simplified into *sap*.

Let *𝛷* be the string associated with a specific simulation instance as described in the previous paragraph. We first compute the following 5 parameters: [*p_1_*] is the Levenshtein similarity [44] between the string consisting of the first 3 characters of *𝛷* and the reference string *sap* as we have assumed that the expected path in collecting clinical data is going from the input scenario to the history taking and then to the physical examination [45,46]. [*p_2_*] is the Levenshtein similarity between the string consisting of the last 2 characters of *𝛷* and the reference string *br*. *b* stands for binary analysis and *r* stands for result or final diagnosis selection. This is done because the expected last steps in a simulated case should be to analyze the collected clinical data to select the diagnostic hypothesis deemed to be correct according to the hypotheticodeductive model [47,48]. [*p_3_*] is a parameter whose value is 1 if the first occurrence of *h* (hypothesis generation) precedes the first occurrence of *m* (medical test); otherwise, it is 0. Indeed, we assumed that medical tests should only be requested after at least one diagnostic hypothesis is formulated [49], also according to the *choosing wisely* campaign [50]. [*p_4_*] is the percentage of sections present in *𝛷* out of the 7 possible sections. Hence, for instance, if *Φ= sapr,* then this parameter is 4/7. This is to ensure that the student makes a comprehensive assessment of the simulated patient without missing any sections of the case. [*p_5_*] *is* the parameter 1/(1 + *R*), where *R* is the number of repetitions in *𝛷*. This is to favor a linear approach to the case over a repeated back-and-forth movement throughout the sections as it may occur with less proficient diagnosticians [35,36] possibly prone to premature closure [51,52].

These 5 parameters are then combined into a single score by computing the Euclidean norm of the vector whose dimensions are the 5 parameters:

√(p_1_^2^ + p_2_^2^ + p_3_^2^ + p_4_^2^ + p_5_^2^).

### Metric Validation

Our proposed metrics were preliminarily validated using test 1 results. As the simulated clinical case in test 1 was about Hodgkin lymphoma, to validate our new metrics, we compared the results with the current reference standard to assess students’ knowledge in hematology at our university, that is, the hematology curricular examination. This examination consists of a multiple-choice question test on hematologic disease epidemiology, risk factors, clinical presentation, and diagnosis. The score ranges from 0 to 33.

For validation, we compared the overall, collection, analytical, and methodological scores with the hematology examination score using the Spearman rank correlation test.

## Results

### Overview

**Figure 1:**
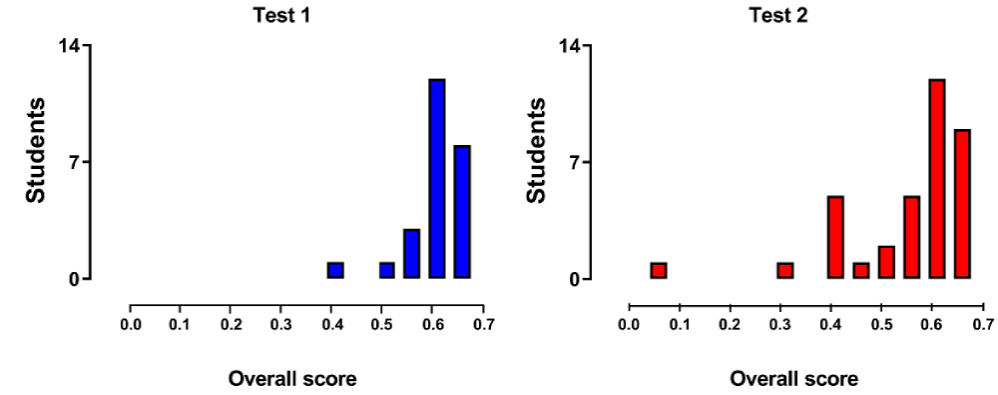
Class overall performance scores during tests 1 and 2 as shown by histogram bar distribution. During test 2, the presence of bars on the left side points to the existence of students characterized by a weaker overall performance compared with the rest of the class. The range of each bar is 0.05.

The average class performance was slightly greater for test 1 (mean 0.59, SD 0.05) than for test 2 (mean 0.54, SD 0.12), with a larger score dispersion during test 2 as suggested by the greater SD. Figure 1 shows the class performance as assessed by the overall score distribution obtained during tests 1 and 2. The overall scores were not normally distributed, as evidenced by the left-skewed bars. This suggests that a minority of students performed worse than the class average, particularly during test 2.

**Figure 2:**
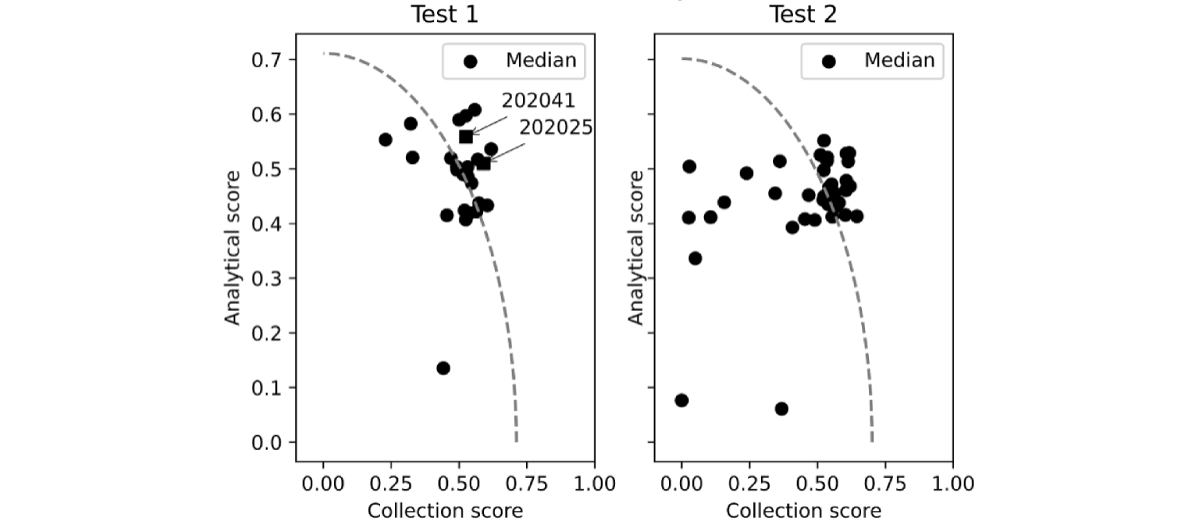
Relationship between collection and analytical scores during test 1 (April 12) and test 2 (May 21). Each dot represents the performance of a single student. The ideal (maximal) performance score corresponds to 1.0. The dashed line indicates the median of the overall scores of the class. Note that students 202025 and 202041 (arrows) reached a similar overall score (0.63) in different ways. Student 202041 performed worse in the data collection exercise (collection rank 12 and analysis rank 5), whereas student 202025 performed poorly in the data analysis exercise (collection rank 3 and analysis rank 11) compared with the class results.

By grouping the 7 metrics into 2 knowledge domains (ie, data collection and data analysis; Figure 2), we could gain further insights into the students’ expertise. Note the different dispersions of single scores during the 2 tests. The greater cluster of single scores during test 1 points to a more homogenous class performance. In addition, if only the overall performance scores were considered, students 202025 and 202041 (see arrows), for example, would appear to be at the same performance level. However, in their case, the identical overall scores (0.63) were reached in a different manner: student 202041 performed worse on the data collection exercise (collection rank 12 and analysis rank 5), whereas student 202025 performed poorly on the data analysis exercise (collection rank 3 and analysis rank 11).

**Figure 3:**
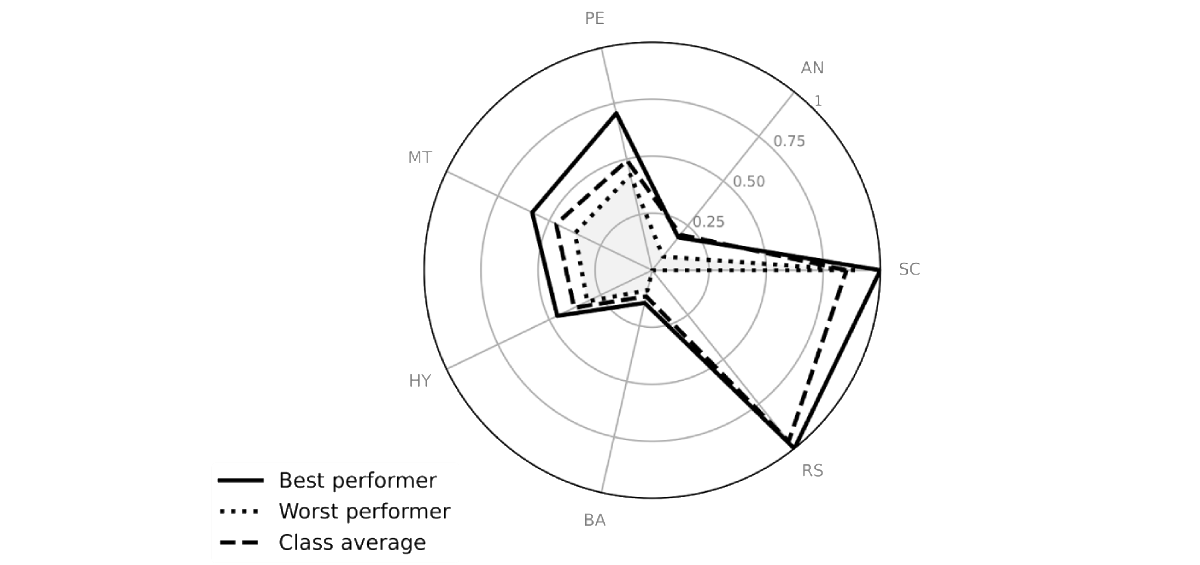
Radar graphs of the top- and bottom-performing students and average class results in each exercise section during test 1. Graphs enabled the comparison between the scores of the different exercise sections of the simulation as obtained by the top (continuous line) and bottom (long dashed line and grey area) performers and by the class (short dashed line). Note that the top-performing student scored consistently better than the average of the class on all tasks except the history-taking exercise. In contrast, the bottom performer scored less in every exercise except the anamnesis. The 2 students could be given individualized advice by teachers to overcome each specific weakness. The results refer to test 2. AN: anamnesis; BA: binary analysis; HY: hypothesis generation; MT: medical tests; PE: physical examination; RS: results; SC: scenario.

Further analysis of student performance may be obtained using radar charts, as shown in Figure 3. In every diagram, the scores obtained in each of the 7 simulation sections can be summarized and compared with the performance of other students to detect the topics in which the student needs improvement.

Note that the top-performing student scored consistently better than the class average in all tasks except in the history-taking section. Conversely, the bottom performer reached the class average level only in the identification of relevant information within the given clinical scenario task (ie, interpretation of the input scenario).

**Figure 4:**
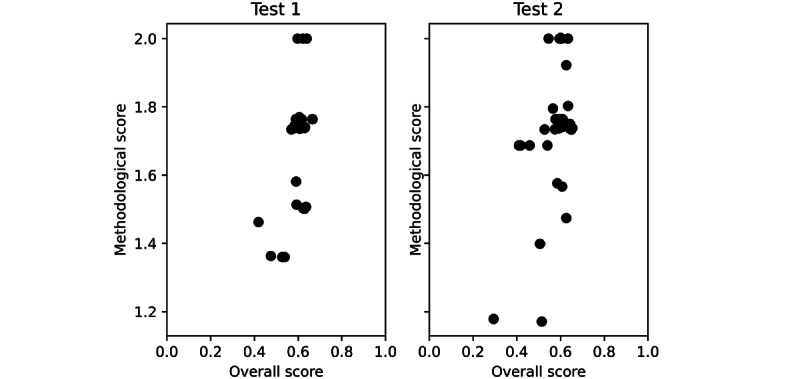
Relationship between individual overall scores and corresponding methodological scores obtained during test 2. The arrow indicates the students who scored weakly as far as the clinical methodology is concerned, although the overall score was acceptable. Therefore, this student is specifically lacking in their way of addressing that diagnosis and needs ad hoc teacher’s advice.

Figure 4 provides insights on the students’ skills in clinical methodology. The methodological score obtained by each student during tests 1 and 2 was plotted in relation to the overall score. The arrow indicates the student who scored poorly during test 2 as far as the clinical methodology was concerned despite an acceptable overall score.

**Figure 5:**
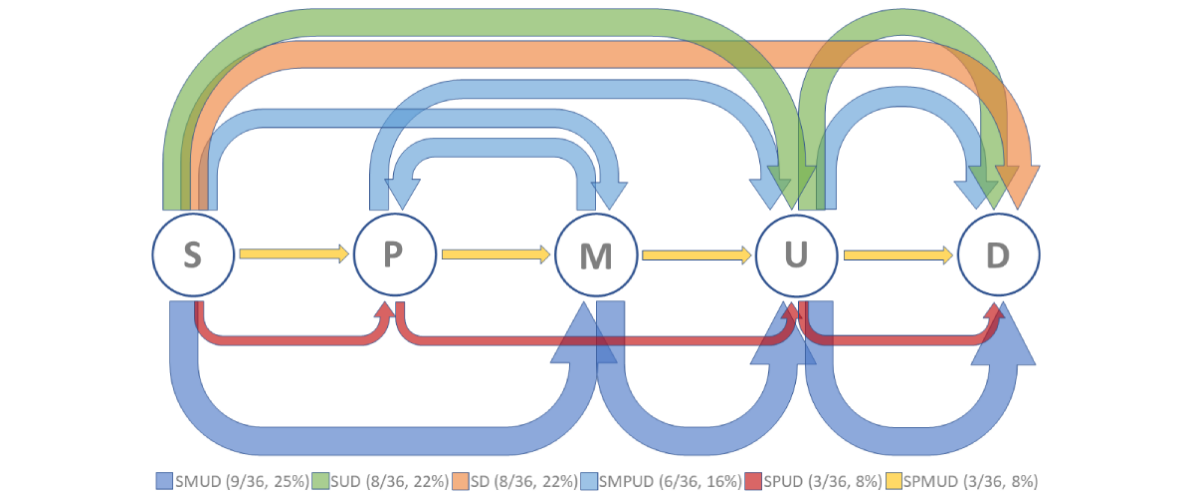
Critical diagnostic acts and expected execution path during test 2. The most likely diagnosis in that simulation was cholecystitis, and the key actions the user was expected to perform from the start (S) were previously set to be (1) palpation of the abdomen (right upper quadrant; P), (2) check for the Murphy sign (M), (3) request for abdomen ultrasonography (U), and (4) final diagnosis (D), corresponding to the PMUD pathway (thin yellow arrow). Each arrow represents a different execution flow. The width of the arrow is proportional to the number of students who followed that flow. Note that, of the 36 students, only 3 (8%) executed all 3 crucial diagnostic acts in the expected order, whereas 16 (44%) reached the correct final diagnosis without performing a physical exam, and 8 (22%) gave priority to abdomen sonography.

Figure 5 displays the sequences of the crucial diagnostic acts that were performed by the students during the test 2 simulation and the number and percentage of users who performed each sequence. The 5 crucial diagnostic acts for test 2 were analysis of the input scenario (S), palpation of the abdomen (P), search for the Murphy sign (M), request for an abdominal ultrasound (U), and selection of the correct final diagnosis (D). Of the 36 students, only 3 (8%; SPUD) executed all 3 crucial diagnostic acts in the expected order, whereas 16 (44%) reached the correct final diagnosis without performing a physical examination.

### Metric Validation

Of the 25 students who took test 1, 20 (80%) disclosed their hematology examination scores. Of those 20 students, 1 (5%) scored 25, 6 (30%) scored 29, and the remaining 13 (65%) scored 33. As reported in Table 2, there was no correlation between the hematology examination score and each of the Hepius metric scores.

**Table 2:** Results of the Spearman rank correlation test between the hematology examination score and the 4 main Hepius metrics.

Metric	Correlation index	*P* value
Overall score	0.2867	.22
Collection score	0.2786	.23
Analytical score	−0.0404	.87
Methodological score	0.0836	.73

## Discussion

### Principal Findings

In this paper, we describe the learning analytics obtained using the VPS Hepius [23] by analyzing the results of 2 tests performed by fifth-year students of the International Medical School at Humanitas University. In addition, learning analytics were preliminarily validated by comparing them with the hematology curricular examination score during test 1.

Learning analytics may provide teachers with valuable information on students’ medical expertise and diagnostic reasoning skills. However, *remediable* should be the desired key feature of an education performance metric, in the specific sense of being suitable for remedial actions. Not all metrics have this characteristic, and most are designed only for evaluative purposes. For instance, the examination score is a global indicator of competence in a specific area and provides limited direct hints on what the student should focus on to improve competence. Evaluation, rather than remediation, is the primary goal of an ordinary examination score [53-56]. In contrast, the main metrics presented here (ie, overall score, collection score, analytical score, and methodological score) were developed primarily to provide educators with clues on student-centered *remedial actions*.

In this study, we first set basic statistical metrics to assess students’ performance on single sections of the simulations. By combining these metrics, a convenient index (ie, the overall score) was computed featuring the students’ global performance. In addition, relative graphs were drawn to synthesize the main results.

Much information is provided by such an analysis (Figure 1) and can be grouped as follows: (1) in-class information; for example, the left-sided bars in Figure 1—the test 2 histograms suggest that there are students who performed worse than most of the class; from an educational standpoint, this subgroup of students may be the target of specific teaching actions aimed at *sliding them to the right side of the graph*—and (2) cross-class information (eg, a comparison between the same classes of different academic years), which may provide teachers with information concerning their overall teaching performance over time.

Another valuable issue is the possibility of comparing student and class performances using the radar chart. This summarizes the single scores obtained during the different exercises of the simulation. Radar charts can be drawn for a single student or the entire class performance. In Figure 3, the top-performing student scored better than the rest of the class in all exercises but 1 (ie, history taking). This may reflect an overconfident behavior of the *smart* student who, having intuitively interpreted the clinical case using little information, did not deepen into the history taking, thus losing important information and falling into what is called an “early closure mistake” [57]. From a didactical point of view, each result obtained from simulations may provide specific insights on the overall class competence level and on specific features of each student’s knowledge at the same time.

The overall score may provide information on the capability of the student to accurately analyze the clinical case. If appropriate strategies were used to avoid laziness and strict time bounds were preset, we might expect the overall score to be a proxy measure of the examination score as far as the related topics are concerned, although the results of this study do not support such a hypothesis.

However, the overall score would not be expected to be particularly useful as a remedial tool. Conversely, by making correlations between the 2 components of the overall score (ie, the data analysis and data collection scores), important operative information on students’ diagnostic process could be obtained. For example, it is possible to assess the relative contribution of data analysis or data collection scores to the individual overall score, potentially giving the student specific advice to overcome any weakness. In addition, students who have an unsatisfactory analytical score should focus their attention on learning the specific UpToDate [58] documents automatically suggested by the Hepius Learner Model or enhancing their expertise in specific diseases through medical literature revision. In contrast, those with unsatisfactory data collection scores should exercise more with Hepius clinical cases or by directly interviewing real patients. Notably, such a hypothesis has not yet been validated and requires an ad hoc study.

The methodological score we propose aims to estimate the extent to which a student follows an adequate and realistic diagnostic process. We trust in clinical methodology and believe that its main principles must be learned by medical students [41-43] despite the recent widespread attitude in favor of using technologies for diagnostic purposes. A proper methodological approach to patients, both diagnostic and therapeutic and possibly evidence-based, may optimize diagnosis [59] and therapy [60] while diminishing the side effects [61,62]. Moreover, such a *choosing wisely* approach may eventually affect health care costs by remarkably reducing unnecessary tests, examinations, and treatments [63-66]. In a simulation of acute cholecystitis, by identifying the diagnostic actions performed and tracking the sequence of their execution, the use of Hepius revealed that, in the process of reaching the final diagnosis, >40% of the students (16/36, 44%) skipped the abdominal physical examination, and 22% (8/36) went straight to perform an abdominal ultrasound (Figure 4). There are 2 possible explanations for this finding. It might be because the students were dealing with a virtual simulation rather than a real patient on whom they would actually perform a complete abdominal physical examination. Alternatively, this finding may mirror students’ overdependency on medical tests as a result of low confidence in their diagnostic self-capabilities. In both cases, an important educational challenge is posed requiring both recognition and properly targeted teaching action. An example of the latter would be a teacher referring a student with an insufficient methodological score to appropriate guidelines or flowcharts addressing the specific management of the disease or disorder.

In keeping with these considerations, we also sought to assess the magnitude of the methodological component within the individual student overall score by initially setting the 2 scores and then plotting the methodological score versus the overall score. As shown in Figure 4, some students performed quite poorly in clinical methodology [67] despite an acceptable overall score. In fact, their overall scores were close to the class average. Therefore, such an approach enabled us to identify students who could have taken learning advantages if promptly referred by the program or the teacher to an adequate UpToDate chapter or disease management guidelines.

Addressing cognitive processes using simulators is a daunting task that has been automatically approached in different ways. For example, Hege et al [68] used a VPS combined with a concept mapping tool to assess a number of actions performed by students, including problem identification, differential diagnosis setting, test requests, treatment options, and connections made. Similarly, Hepius can track the interactions between students and the simulator and synthesize them in a fuzzy cognitive map. Unlike the tool used by Hege et al [68], Hepius may identify the crucial diagnostic acts and their execution order without focusing on the diagnostic accuracy, defined as the capability to reach a correct final diagnosis on the first attempt [67]. We assumed a priori that, for every symptom, there was a set of fundamental actions that a student should take to reach a proper diagnosis. Importantly, the right order of actions was also essential as it may simplify the diagnostic pathway without the need for unnecessary tests [63,64]. Finally, we hypothesized that identifying these actions and their execution order within the simulation could be used as a proxy, possibly reflecting the students’ overall cognitive process and methodological skills. Although all students (36/36, 100%) could reach a correct final diagnosis, our data suggest that only 8% (3/36) of them followed the desired sequence (the SPMUD path in Figure 5), which was assumed to be methodologically correct, whereas the rest adopted 5 different approaches. Through our simulator, we were able to identify students who omitted critical actions, indicating flaws in their methodological approach toward the patient that could potentially be amended through remediation actions such as learning specific management pathway guidelines.

When comparing overall, collection, analytical, and methodological scores with the students’ hematology examination scores, we found no statistically significant correlation. However, this was expected as the scores addressed different skills [53]. The multiple-choice question examination evaluated global and in-depth competence regarding diseases. The VPS scores aimed to assess the students’ ability to *collect* clinically relevant information (ie, collection score), *formulate a differential diagnosis* from scratch, *make proper connections* between the diagnostic hypothesis and collected clinical information (ie, analytical score), and solve the clinical case using a *proper clinical methodology* (ie, methodological score). Furthermore, it should be noted that the hematology examination scores were quite homogenous as 65% (13/20) of the students scored 33 out of 33 and 95% (19/20) scored >29. Although this may reflect a homogeneous education level of the class, it may also indicate a potential limitation of that evaluation method in properly grasping the wide variability of medical students’ preparation [54-56].

Indeed, although multiple-choice question tests currently represent the mainstay of medical student evaluation, many have highlighted the weaknesses of such an evaluation tool [69,70].

### Limitations

These results were obtained using 2 tests and a limited number of participants. This dampens the generalizability of the results on Hepius’s effectiveness as a tool for the evaluation of medical students’ diagnostic skills. In addition, our proposed learning analytics should undergo a more robust validation, possibly through psychometric methodology [29]; however, this would require a larger student population. The psychometric features characterizing the learning analytics proposed in this study are highlighted and discussed in Multimedia Appendix 1.

### Conclusions

The use of Hepius by fifth-year medical students enabled us to obtain valuable educational information that was organized according to the proposed learning analytics. Insights obtained using learning analytics might better guide the teacher’s feedback aimed at filling students’ gaps in both medical knowledge and diagnostic methodology. It is important to highlight that Hepius learning analytics might also be used in different postgraduate settings, such as for the yearly assessment of residents’ clinical training and general practitioner preparation within the continuing medical education context.

Ad hoc future studies are required to fully validate our proposed learning analytics.

## Appendix

Multimedia Appendix 1 Hepius learner analytics psychometric features.

## Abbreviations

ITS: intelligent tutoring system
NLP: natural language processing
SCT: script concordance test
SNOMED–CT: Systematized Nomenclature of Medicine–Clinical Terms
VPS: virtual patient simulator
