# Supplementary material for: Learning Analytics Applied to Clinical Diagnostic Reasoning Using a Natural Language Processing–Based Virtual Patient Simulator: Case Study
Source: JMIR Med Educ. 2022 Mar 3;8(1):e24372. doi: 10.2196/24372 (PMC8931645; doi:10.2196/24372)
Supplement: Multimedia Appendix 1 [file mededu_v8i1e24372_app1.docx]

# “Hepius learner analytics psychometric features”.

The relationship between Hepius binary analysis and a Script Concordance Test.

Binary Analysis is conceptually close to a Script Concordance Test. According to Fournier et al [26], a Script Concordance Test (SCT) consists of the following main blocks: a. A clinical case. b. A diagnostic hypothesis. c. An ‘if you were thinking of’ text block. d. An ‘And then you were to find’ text block. e. “This hypothesis would become” 5-anchor Likert-type scale. In Hepius, item a. is provided by the teacher as an initial scenario; b. is formulated by the student (diagnostic hypothesis generation); c. and d. are the diagnostic factors identified by the student; and e. is the actual content of the Binary Analysis, but with a 3-anchor scale. Hence, if we focus on the Binary Analysis of a single diagnostic hypothesis, the Binary Analysis is a SCT with a 3-anchor scale. The key differences are that in Hepius: 1. the diagnostic hypothesis is formulated by the student, rather than being provided a priori in the test; 2. there may be multiple diagnostic hypotheses; 3. the diagnostic factors are gathered by the student, rather than being formulated a priori; 4. we split the analysis work in two phases: I. In the Binary Analysis the student uses a 3-anchor scale II. In the Pattern Analysis the student can refine the analysis with a greater granularity scale. We point out that in I. the student receives feedbacks, while in II. the student does not receive any feedbacks. The rationale for the I.- II. split is that the inter-rate agreement for the 3-anchor scale is far greater than that for the 5-anchor scale. Moreover, experience has shown that such a split makes the exercise easier to be completed by students.

Pattern Analysis, Cognitive Fuzzy Maps and Concept Mapping

In the Pattern Analysis, the student creates a Cognitive Fuzzy Map (CFM) relating DFs to DHs. The choice of using CFMs, rather than using other mathematical models such as probabilistic graphs, is because the student is only requested to establish qualitative (i.e., fuzzy) estimates of the strength of the association between DF and DH. This is in agreement with the fact that physicians tend to rely primarily on qualitative evaluation for diagnosis rather than on precise quantitative assessments, as the information upon which medical diagnosis is usually based is intrinsically uncertain. Indeed, fuzzy logic has been extensively used for modeling computer-assisted medical diagnosis systems [30].

For educational purposes, we decided to adopt a simplified version of a CFM for the Pattern Analysis. In this setting the connecting edges may only go from a diagnostic factor to a diagnostic hypothesis and there are no edges between diagnostic factors or between diagnostic hypotheses. Indeed, in its current implementation, the Pattern Analysis graphs are bipartite graphs with two types of nodes (diagnostic factors and diagnostic hypotheses) in which edges can only connect one type of node to the other type of node. The use of non-bipartite graphs makes sense conceptually, as it could enable modeling the interaction between nodes of the same type. For instance, an edge connecting two diagnostic factors is conceptually legitimate and can be handled by Cognitive Fuzzy Maps [28]. A decision was taken to initially work only with simplified models.

It is important to point out that thinking of Pattern Analysis only in terms of bipartite graphs is highly reductive. The Cognitive Fuzzy Maps are graphs in which weights are assigned to nodes and edges. Indeed, the main activity of the student working with these maps during Pattern Analysis is to tune the weights of diagnostic factors nodes and diagnostic hypothesis edges, whereas Cognitive Fuzzy Maps computes the weight of diagnostic hypotheses. Unlike conceptual maps that are static and schematic representations of the possible link between a hypothesized diagnosis and a diagnostic factor, Pattern Analysis requires the learner’s active action in modifying the nodes and arches weights, thus changing the relevance of a diagnostic factor in supporting a certain diagnosis. We believe that this process is inherently characterized by a significant didactical valence. Furthermore, it is automatically translated into a change in the magnitude of the map corresponding to the likely diagnosis. This, in turn, gives the student an additional learning hint.

Psychometric features of learner analytics

# We believe that Hepius [23] can be considered as a novel psychometric tool. In keeping with Cook and Beckam’s approach [29], validity sources of a psychometric tool are based on content, response process, internal structure, relations to other variables and consequences, and reliability. These items are variously addressed both in the present paper and in our recently published study [23]. The following comments will briefly address our Virtual Patients Simulator Hepius mean features in the light of Cook and Beckam’s [29] perspective concerning the main characteristics of a psychometrical tool.

# Content evidence of validity can be found in the appendix 3 of our recent JMIR Med Inform paper [23] where a detailed description of the clinical case creation by the teacher in charge is provided.

# Correctness of the diagnostic findings and steps to be followed by the student (content) are granted by the simulation authoring process that consists of the following main steps: a. one MD (author) prepares the simulation b. two or more MDs (reviewers) review the simulation and provide feedback; c. the author corrects the simulation according to reviewers’ feedbacks; d. the simulation is used by a group of students and feedbacks are collected; e. the simulation is tuned, according to the student feedbacks, by the author after discussion with the reviewers. Presently, this is the best evidence of content validity we can provide about our diagnostic tool.

# Response Process validity is defined as the review of thoughts and actions the student is taking when addressing the psychometric tool content. Response Process validity is a feature intrinsically present in Hepius. Indeed, the log feature described in JMIR Med Inform paper [23] enables to save every action a student makes while addressing the clinical simulation. Using the log as a starting point we can evaluate the diagnostic path the student has followed, as exemplified in figure 5 of the present article. Therefore, it is possible to analyze each action of a student engaged with the clinical case.

# Internal structure validity evidence is represented by the capability of the metrics to capture the heterogeneity of constructs in the assessed population. Indeed, the underlying composite construct (e.g. clinical reasoning) we are attempting to measure is multidimensional and therefore requires sub-scores addressing its different parts. An example is provided by radar charts where differences between charts represent the heterogeneity of the composite construct (figure 3). Reliability is another source of internal structure validity and is addressed separately (see below).

# Validity evidence in relation to other variables is addressed in the Result and Discussion sections of the present manuscript. Metrics we develop did not correlate with multiple-choice questions test, although performed on a content that was a priori supposed to be similar. The lack of correlation supports the hypothesis that the construct measured by our metric is different from the construct measured by multiple-choice questions test. Indeed, the multiple-choice test measures the knowledge of a specific subject whereas our psychometric tool assesses the clinical reasoning applied to the same issue. In keeping with Cook and Beckman [29] perspective, we have to admit that validity evaluation is an ongoing process of testing and revision. This process is far from being completed with regards to our novel tool.

# Evidence of consequence was indirectly taken into account in the current study. Indeed, in the methods and discussion sections we emphasize that our psychometric tool is designed to collect information dealing with the student’s activities during a diagnostic task. Each student’s tracked action can be used by the teacher for setting remedial suggestions aimed at overcoming any specific diagnostic weakness. Potentially, these metrics enable the identification of flaws both in the diseases knowledge and simulated clinical practice. This permits a wide variety of remediation actions. In addition, the use of our psychometric tool has shown to effectively improve student knowledge in the short-term, as seen in the results of our recently published paper [23]. Notably, effectiveness of remedial actions has yet to be fully assessed.

# Regarding reliability, Hepius seems to act as a reliable psychometric tool. In this context, the key aspect is that Hepius is a computer-based technology, thus ensuring temporal stability and inter-rater agreement. Indeed, yielded results depend on calculations related to the correctness of student’s action and are independent of an external evaluator’s judgment. Furthermore, the use of multiple scores permits the measurement of the different aspects of the underlying construct, thus meeting the requirements of internal consistency.

# 
